# Preoperative risk stratification of mediastinal masses using a predictive model based on CT radiomics and clinical data

**DOI:** 10.3389/fsurg.2026.1937652

**Published:** 2026-09-17

**Authors:** Kun Li, Xiangyu Xie, Yi Fei Feng, Lei Chen, Liang Zheng

**Affiliations:** 1 Department of Thoracic Surgery, The First People’s Hospital of Changzhou and The Third Affiliated Hospital of Soochow University, Changzhou, China; 2 Department of Thoracic Surgery, Xuzhou Central Hospital, Southeast University, Xuzhou, China

**Keywords:** clinical baseline data, mediastinal tumor, predictive model, preoperative prediction, radiomics

## Abstract

**Objective:**

Mediastinal masses pose substantial diagnostic challenges because of their complex anatomical relationships and heterogeneous pathological characteristics. This study aimed to develop and internally validate a multiparametric predictive model integrating CT radiomics and clinical variables, including the serum tumor markers CYFRA21-1, SCC-Ag, and CA19-9, for preoperative stratification of mediastinal lesions into high- and low-risk categories according to their invasive or malignant potential.

**Methods:**

This single-center retrospective study included 157 patients who underwent surgical resection of mediastinal tumors at the Third Affiliated Hospital of Soochow University between January 2010 and October 2024. Clinical, imaging, and laboratory data were collected, and two radiologists independently delineated and evaluated the lesions on preoperative contrast-enhanced CT images. Patients were randomly allocated to a training cohort (*n* = 110) and an internal validation cohort (*n* = 47). A radiomics model was developed using least absolute shrinkage and selection operator (LASSO) regression to select informative features from 852 extracted radiomics features. An integrated model was subsequently developed by combining the radiomics score with relevant clinical variables, including tumor size, CYFRA21-1, SCC-Ag, and CA19-9. Model performance was evaluated in the training and internal validation cohorts. No external validation cohort was available.

**Results:**

In the training cohort (*n* = 110), the integrated model achieved an area under the curve (AUC) of 0.9117 (95% CI: 0.8586–0.9648) with a sensitivity of 85.25% and a specificity of 81.63%, compared to an AUC of 0.8985 (95% CI: 0.8407–0.9562) for the radiomics-only model. In the internal validation cohort (*n* = 47), the integrated model maintained stable diagnostic performance with an AUC of 0.8718 (95% CI: 0.7739–0.9697), a sensitivity of 84.62%, and a specificity of 71.43%, whereas the AUC for the radiomics-only model was 0.8599 (95% CI: 0.7488–0.9709).

**Conclusion:**

A model integrating radiomics and clinical data may serve as a potentially effective tool for preoperative risk stratification of mediastinal tumors; this model shows great promise for optimizing individualized surgical planning. However, as this study was a single-center, retrospective design and lacked external validation, the model's generalizability requires further confirmation through large-scale, multicenter prospective studies.

## Introduction

Mediastinal tumors are an important category of space-occupying lesions within the thoracic cavity and are clinically significant due to their complex anatomical and pathological characteristics. Mediastinal tumors are relatively rare. Population-based data on the overall incidence of mediastinal tumors are limited because mediastinal masses comprise a heterogeneous spectrum of benign and malignant entities. Recent studies based on chest CT examinations have reported that incidentally detected mediastinal masses occur in approximately 0.73%–1.68% of examined individuals (1–3). These lesions exhibit considerable heterogeneity in biological behavior, spanning a spectrum from benign or indolent conditions to highly invasive or frankly malignant tumors. Consequently, accurate preoperative risk stratification is clinically essential, as therapeutic management—including surveillance, minimally invasive intervention, or definitive resection—is critically dependent on the anticipated degree of biological aggressiveness, local invasiveness, and malignant potential (2). Given their diverse clinical presentations, establishing a comprehensive diagnostic system that integrates multimodal imaging with molecular pathology is crucial for achieving precise staging, guiding personalized treatment, and improving long-term prognosis (4, 5).

Radiomics, as an emerging technique for quantitative analysis of multimodal medical imaging, can extract high-dimensional features from CT, MRI, and PET scans, including textural heterogeneity, morphological parameters, and metabolic indicators (6, 7). Although radiomics has increasingly been investigated for the characterization of mediastinal lesions, previous studies have largely focused on differentiating specific pathological entities, such as anterior mediastinal masses, thymomas, and thymic cysts, rather than on clinically oriented risk stratification across a heterogeneous spectrum of mediastinal tumors (8, 9). While AI-based diagnostic models have been developed, these models typically rely on imaging data or limited clinical parameters, neglecting the valuable molecular information provided by serum tumor markers—which are independent of imaging morphological features. This limitation hinders the models' ability to comprehensively capture the biological behavior of tumors and may lead to failure to identify aggressive tumors with atypical imaging features, such as small thymic carcinomas (10). Furthermore, these models often neglect key clinical risk factors—such as tumor size, location, serum tumor markers, and the patient's overall health status—making it difficult to assess the interactions among complex variables (11).

With continuous advances in imaging technology, radiomics has evolved into an advanced system capable of overcoming the limitations of traditional diagnostic methods. This technology can extract biologically meaningful parameters that reflect tumor heterogeneity at the subcellular level—a factor closely associated with tumor invasiveness, malignancy, and prognosis (12). Despite these advances, systematic studies evaluating radiomics for preoperative risk stratification of heterogeneous mediastinal lesions according to invasive or malignant potential remain limited.

Although radiomics has been increasingly applied to the characterization and risk assessment of mediastinal lesions, most previous prediction approaches have primarily relied on imaging-derived features or a limited set of conventional clinical variables. Therefore, the principal novelty of the present study does not lie in the application of radiomics alone, but rather in the multimodal integration of quantitative CT radiomics features with serum tumor biomarkers that reflect complementary biological information. Specifically, we incorporated the radiomics score together with serum CYFRA21-1, SCC-Ag, and CA19-9, as well as tumor size, into a unified predictive framework for the preoperative stratification of mediastinal lesions. By combining imaging-derived phenotypic information with circulating biomarker information, this approach was designed to capture complementary aspects of tumor morphology, heterogeneity, and biological behavior that may not be adequately represented by either data source alone. The primary objective of this study was therefore to evaluate the feasibility and potential incremental value of this multimodal integration strategy for distinguishing high-risk lesions with invasive or malignant potential from low-risk mediastinal lesions.

## Materials and methods

### Materials

#### Selection and grouping of patients

This retrospective study enrolled patients diagnosed with mediastinal space-occupying lesions who underwent surgical treatment at the Department of Thoracic Surgery and Cardiothoracic Surgery, Third Affiliated Hospital of Soochow University, between January 2010 and October 2024. Patient selection was based on predefined inclusion and exclusion criteria, which included age range (18–80 years), lesion type (benign or malignant), and availability of preoperative CT imaging. Patients with a history of previous thoracic surgery or other malignancies were excluded from the study.

A total of 157 patients were included in the final analysis, comprising 75 males (mean age: 55.08 ± 11.04 years) and 82 females (mean age: 56.56 ± 12.68 years). The patients were randomly assigned to either a training (experimental) cohort or a validation cohort in a 7:3 ratio. The training cohort consisted of 110 patients, including 49 classified as high-risk, while the validation cohort comprised 47 patients, with 21 identified as high-risk. Randomization was performed using a computer-generated randomization sequence to ensure a balanced distribution of high-risk cases across both cohorts. There was no statistically significant difference in the distribution of high-risk cases between the two cohorts (*P* > 0.05), indicating good baseline comparability.

Low-risk group: Patients whose lesions show limited invasiveness or biologically indolent behavior, including truly benign lesions (e.g., thymic cysts, pericardial cysts, lipomas). In this study, “low risk” is not synonymous with absolute benignity, nor is “high risk” synonymous with complete malignancy; however, the high-risk group includes all pathologically confirmed malignant lesions and WHO type B2/B3 thymomas with potential for invasive behavior (13, 14).

High-risk group: In this study, “high-risk” is defined as having clinically significant invasive growth potential or clearly malignant biological behavior, specifically including: (1) pathologically confirmed malignant lesions, such as thymic carcinoma (including squamous cell carcinoma and neuroendocrine tumors), lymphoma, and malignant germ cell tumors; (2) thymomas classified as WHO histological grades B2 and B3; although these lesions are categorized as thymic epithelial tumors, clinical guidelines recommend radical resection in accordance with the principles for malignant tumors, and they exhibit clear invasive potential in terms of biological behavior. It should be noted that the inclusion of B2/B3-type thymomas in the high-risk group is based on the fact that their clinical management strategies (requiring complete tumor resection and evaluation of postoperative adjuvant therapy) overlap significantly with those of malignant lesions, rather than equating them with “malignant tumors” in the traditional sense. WHO type A and AB thymomas, due to their lower invasiveness, were classified into the low-risk group in this study (13–16).

“Preoperative predictive accuracy” is defined as the consistency between the predictive results (high-risk or low-risk) of a model constructed using preoperative CT and clinical information and the postoperative histopathological assessment (including the invasiveness grade). The diagnostic performance of this model is evaluated using metrics such as sensitivity, specificity, and accuracy, with histopathological diagnosis serving as the gold standard.

#### Inclusion and exclusion criteria for patients

**Inclusion criteria:**

Patients who were deemed eligible for surgical intervention.

Patients with complete postoperative pathological results confirming the presence of mediastinal tumors, including both benign and malignant lesions.

Patients who underwent preoperative chest CT imaging with high-quality, interpretable images and no evidence of other concurrent tumors.

**Exclusion criteria:**

Patients with suboptimal CT image quality that hinders the accurate extraction and interpretation of imaging features.

Patients with missing essential baseline clinical data, such as demographic information, tumor size, and relevant serum tumor markers.

Patients with active infections or acute conditions that may significantly increase the risk of complications during surgery.

Patients with severe comorbidities, such as uncontrolled cardiopulmonary diseases, significant coagulation disorders, or advanced-stage renal disease, which contraindicate surgery.

The patient selection process is illustrated in Figure 1.

**Figure 1:**
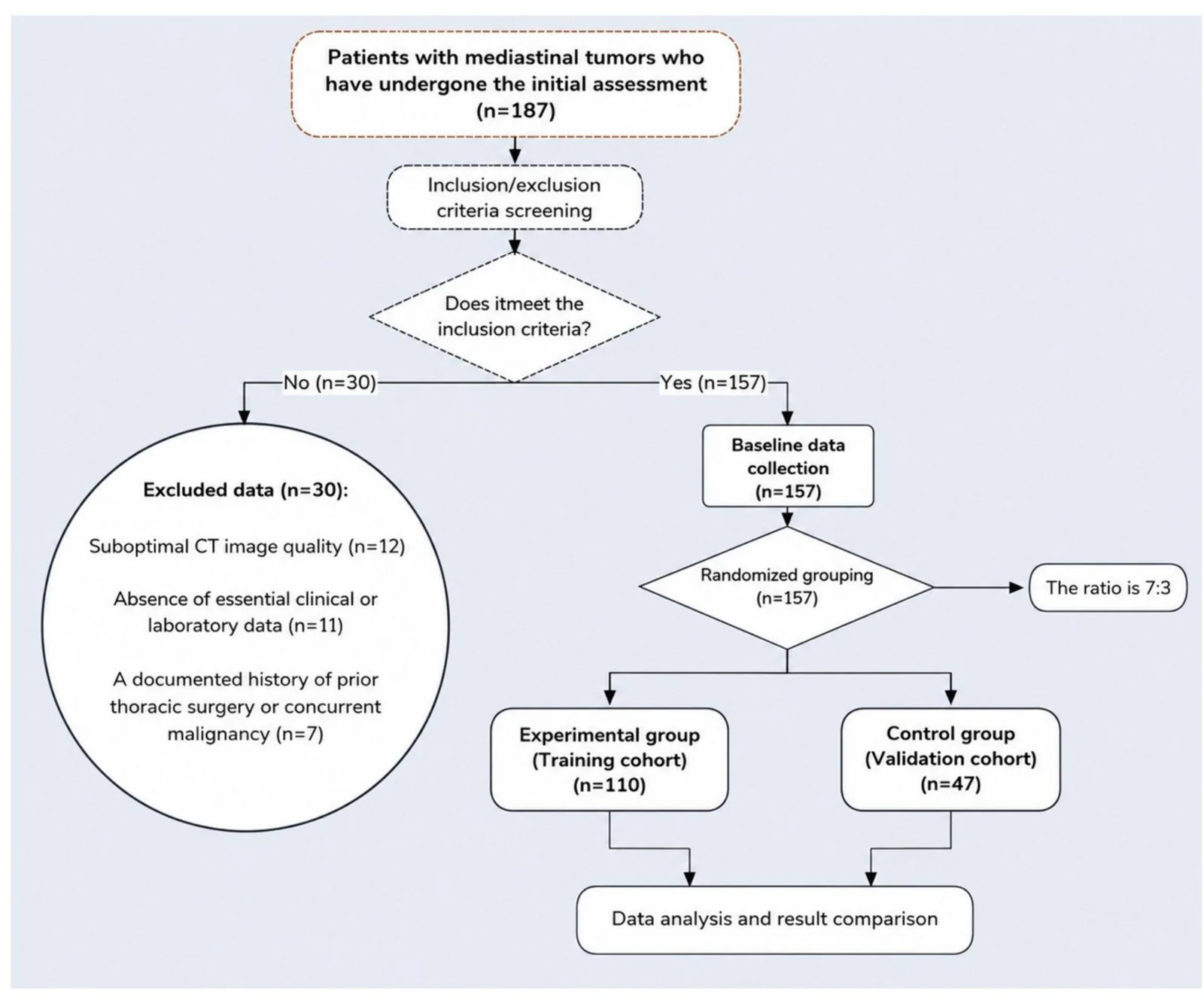
Patient selection nflowchart.

### Methods

#### Treatment of missing data

This study adhered strictly to the predefined inclusion and exclusion criteria. For CT images of insufficient diagnostic quality, these were excluded from analysis based on exclusion criterion 1. The completeness of clinical variables, including tumor markers such as CA125, was verified through the electronic medical record system. Patients with missing values in any essential variable were excluded from the analysis, and no data imputation was performed to preserve data integrity. Continuous variables were screened for outliers using Tukey's method (i.e., values falling outside 1.5 × IQR beyond the first or third quartile); no outliers warranting exclusion or transformation were identified.

#### Method for collecting clinical baseline data

The clinical baseline data of patients were obtained from the medical record system, encompassing the following aspects:

Basic Patient Information: Gender, age, height, weight, BMI, smoking history.
Medical and Surgical History: History of mediastinal tumor resection.
Imaging Data: Maximum tumor diameter, measured from preoperative CT scans.
Pathological Features: Tumor pathological results and lymph node metastasis status.
Tumor Marker Data: Including CA-199, CA-125, CYFRA21-1, NSE, CEA, AFP, SCC-Ag.

#### CT image acquisition and radiomics feature extraction

As this was a retrospective study spanning from January 2010 to October 2024, CT examinations were performed using different CT scanners according to the equipment available at our institution during the respective study periods. During the earlier period (2010–2013), CT examinations were performed using either an Aquilion ONE CT scanner (Canon/Toshiba Medical Systems) or a SOMATOM Sensation 16 CT scanner (Siemens Healthineers). In the subsequent period, examinations were performed using a SOMATOM Force dual-source CT scanner (Siemens Healthineers) after its introduction into routine clinical practice at our institution.

Although CT acquisition protocols evolved over the study period, all examinations included diagnostic-quality chest CT images covering the thoracic inlet to below the costophrenic angles. Patients were examined in the supine position, generally with their arms raised above the head, and were instructed to hold their breath during image acquisition. The detailed acquisition and reconstruction parameters for the different scanners and study periods are summarized in Table 1.

**Table 1:** CT acquisition and radiomics preprocessing protocols used during the study period.

Parameter	Aquilion one	Somatom sensation 16	Somatom force
Manufacturer	Toshiba/canon medical systems	Siemens healthineers	Siemens healthineers
Study period	January 2010–december 2013	January 2010–december 2013	After installation at our institution until october 2024
Scan mode	Helical ct	Helical ct	Helical ct
Tube voltage	120 kvp	120 kvp	100–120 kvp
Tube current	Automatic exposure control (approximately 100–300 mas)	Automatic exposure control (approximately 100–250 effective mas)	Automatic tube current modulation (care dose 4d)
Rotation time	Routine clinical protocol	Routine clinical protocol	Routine clinical protocol
Pitch	Routine clinical protocol	Routine clinical protocol	Routine clinical protocol
Detector configuration	Multi-detector CT	16-row detector CT	Dual-source CT
Reconstruction slice thickness	1–5 mm	1–5 mm	1–3 mm
Reconstruction interval	≤slice thickness	≤slice thickness	≤slice thickness
Reconstruction algorithm/kernel	Standard or medium-smooth soft-tissue reconstruction kernel	Standard or medium-smooth soft-tissue reconstruction kernel	Standard or medium-smooth soft-tissue reconstruction kernel
Contrast enhancement	Contrast-enhanced CT, venous phase	Contrast-enhanced CT, venous phase	Contrast-enhanced CT, venous phase
Contrast agent	Nonionic iodinated contrast medium (300–370 mg i/mL)	Nonionic iodinated contrast medium (300–370 mg i/mL)	Nonionic iodinated contrast medium (300–370 mg i/mL)
Contrast dose	Approximately 1.0–1.5 mL/kg (typically 60–100 mL)	Approximately 1.0–1.5 mL/kg (typically 60–100 mL)	Approximately 1.0–1.5 mL/kg (typically 60–100 mL)
Injection rate	2.5–3.5 mL/s	2.5–3.5 mL/s	2.5–3.5 mL/s
Saline flush	20–30 mL	20–30 mL	20–30 mL
Scan delay after contrast administration	Approximately 60–70 s	Approximately 60–70 s	Approximately 60–70 s
Parameter		Setting	
Image analysis phase		Contrast-enhanced venous phase	
Window setting for ROI delineation		Mediastinal soft-tissue window	
Window width		Approximately 350–400 HU	
Window level		Approximately 40–50 HU	
Image intensity used for radiomics		Original CT Hounsfield units (HU)	
Voxel resampling		Isotropic voxel size of 1 mm^3^× 1mm^3^ × 1 mm^3^	
Image interpolation		B-spline interpolation	
Mask interpolation		Nearest-neighbor interpolation	
Gray-level discretization		Fixed bin width	
Bin width		25 HU	
Feature normalization		*Z*-score normalization before model construction	

Because this was a retrospective study spanning more than 14 years, acquisition parameters varied according to scanner generation, patient body habitus, and the clinical protocol in use at the time of examination. The values shown above represent the protocol ranges used for image harmonization and radiomics analysis; scanner-specific acquisition parameters were verified from the available DICOM metadata whenever possible.

Preoperative CT images of mediastinal tumors were imported into 3D Slicer (version 5.2.2) for image processing and radiomics feature extraction. Regions of interest (ROIs) were manually delineated by two independent radiologists along the tumor margins, with careful exclusion of adjacent normal tissues. The resulting ROIs were subsequently used for quantitative radiomics feature extraction. For tumor segmentation, CT images were reviewed using a mediastinal soft-tissue window (approximately WW 350–400 HU and WL 40–50 HU). Window adjustment was performed when necessary to clearly delineate tumor boundaries. Window features were extracted from the original CT attenuation values (HU) rather than the displayed windowed images.

**ROI Segmentation**:

1. **Segmentation**: The ROIs were manually segmented by two radiologists (each with >5 years of clinical experience) employing 3D Slicer software (version 5.2.2), with the complete extraction workflow illustrated in Figure 2.
2. **Extraction Tool and Standardization:** Feature extraction was performed using PyRadiomics (v3.0.1) via the SlicerRadiomics extension in 3D Slicer (v5.2.2). All extracted radiomics features, image preprocessing algorithms, and nomenclature strictly complied with the Image Biomarker Standardization Initiative (IBSI) guidelines.
3. **Feature Classes and Categories:** A total of 852 radiomics features were extracted, comprising 14 shape features (3D/2D), 18 first-order intensity statistics, 75 high-order texture features derived from original images [including Gray Level Co-occurrence Matrix (GLCM), Gray Level Run Length Matrix (GLRLM), Gray Level Size Zone Matrix (GLSZM), Gray Level Dependence Matrix (GLDM), and Neighboring Gray Tone Difference Matrix (NGTDM)], and 745 wavelet-filtered features obtained by applying 3D Coiflet-1 wavelet transformations (across LLL, LLH, LHH, HHH, etc.) to first-order and texture matrices.
4. **Image Preprocessing:** Voxels were resampled to an isotropic resolution of 1.00 mm^3^ × 1.00 mm^3^ × 1.0 mm^3^ using B-spline interpolation. Intensity discretization was performed using a fixed bin width of 25 HU to ensure calculation stability and reproducibility (17).

**Figure 2:**
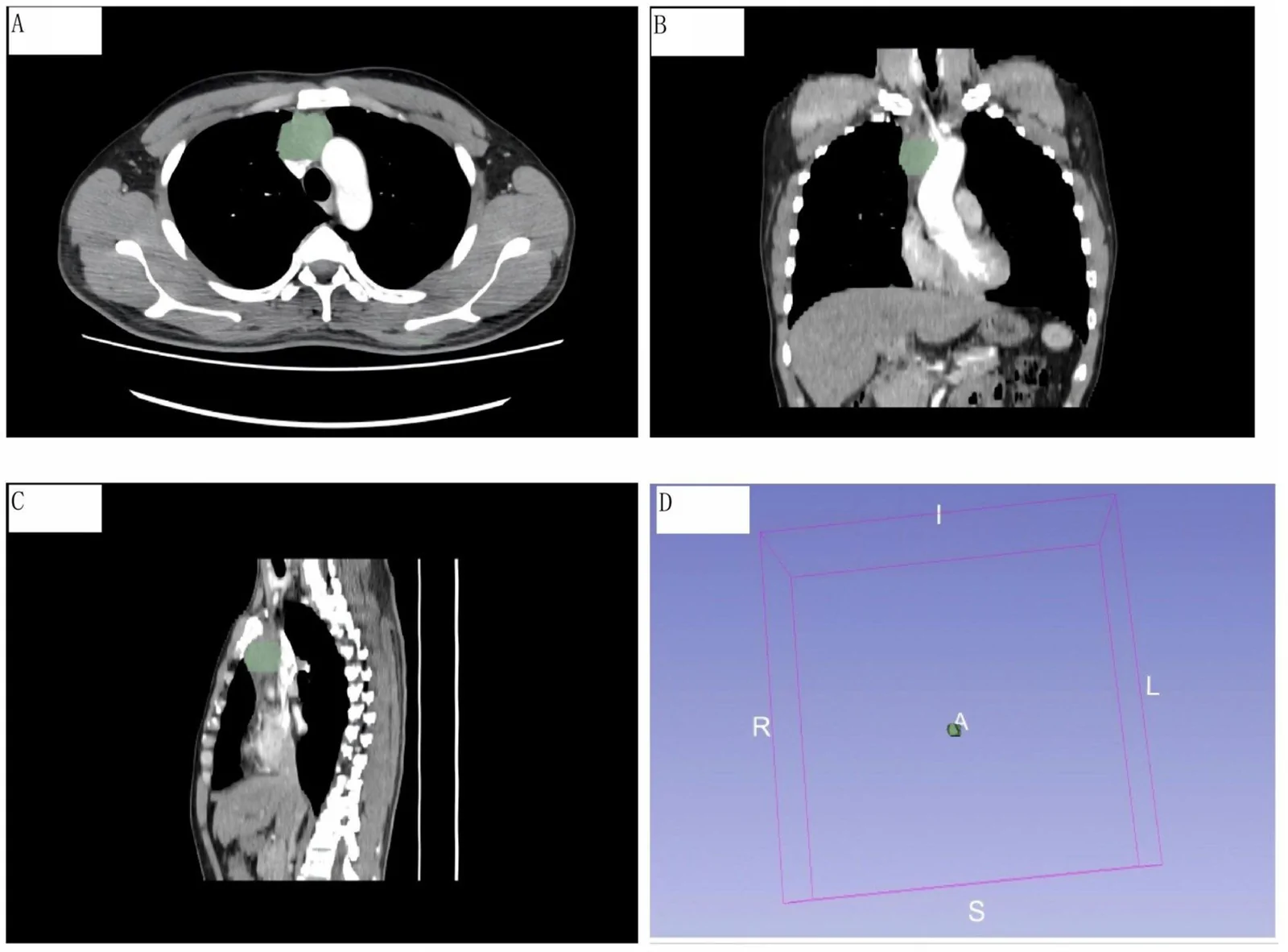
**(A–C)** ROI delineation in the axial, coronal, and sagittal planes. **(D)** Three-dimensional visualization of the segmented ROI.

### Methods for establishing radiomics models and integrated models

#### Use of software

Radiomics feature extraction was performed using PyRadiomics; R was used for statistical analysis and LASSO feature selection, while SPSS was employed for descriptive statistics and binary logistic regression analysis.

#### Establishment of radiomics model

Prior to the extraction of radiomics features, all CT images underwent standardized preprocessing: (1) Voxels were resampled to an isotropic resolution (1.0 mm^3^ × 1.0 mm^3^ × 1.0 mm^3^) using spline interpolation to eliminate spatial resolution differences caused by varying scan parameters; (2) Gray-scale normalization was applied to map CT values to the standard range (−1,000 to +1,000 HU), thereby eliminating the influence of inter-scanner variations in gray-scale distribution on feature values; (3) B-spline methods were used for gray-scale binning, with a fixed bin width of 25 HU, to ensure the stability and reproducibility of texture feature calculations.

To improve model efficiency and reduce the risk of overfitting, all feature preprocessing and selection procedures were performed exclusively within the training cohort (*n* = 110). The mean and standard deviation used for *Z*-score normalization were calculated based on the training cohort only and subsequently applied unchanged to the validation cohort (*n* = 47). Within the training cohort, the 852 radiomics features were first *Z*-score normalized, followed by univariate analysis to preliminarily screen for statistically significant features (*P* < 0.05). Continuous variables were analyzed using *t*-tests, while categorical variables were evaluated using chi-square tests. The selected features were subsequently entered into a LASSO regression model for dimensionality reduction and feature selection. The optimal regularization parameter (*λ*) was determined using 10-fold cross-validation performed exclusively within the training cohort. The validation cohort was not involved in feature selection or parameter tuning and was used only for the final evaluation of the established model. All of the aforementioned feature selection steps–including univariate screening, LASSO regression-based dimensionality reduction, and determination of the optimal *λ* parameter—were strictly confined to the training cohort (*n* = 110). The validation cohort (*n* = 47) was not involved in any form of parameter tuning or feature selection throughout the entire process of feature screening and model development; it was used solely to validate the diagnostic performance of the final model after its training was completed.

##### Establishment of the integrated model

To develop the integrated predictive model, significant clinical variables identified through univariate analysis were combined with the radiomics score (Radscore). These variables were incorporated into a multivariable binary logistic regression model using SPSS 25.0. The model was developed by selecting variables with *P* < 0.05 from univariate analysis and considering their clinical relevance. This multi-level modeling approach, integrating both clinical and radiomics data, was designed to improve the accuracy and clinical applicability of preoperative risk stratification of mediastinal tumors.

**Model Performance Evaluation:**

Model performance was assessed using several metrics:

ROC Curve and AUC Value: The ROC curve visually presents the relationship between sensitivity and 1-specificity at different thresholds, allowing evaluation of model classification ability. The area under the ROC curve (AUC) ranges from 0.5 to 1, with higher values indicating better classification performance. The AUC and its 95% confidence interval were calculated using the DeLong method as implemented in GraphPad Prism.
Sensitivity and Specificity: Sensitivity measures the proportion of true positive cases identified by the model, reflecting the model's ability to detect high-risk cases. Specificity indicates the model's efficiency in identifying true negative cases, with higher specificity values reducing the likelihood of misdiagnosis, thus minimizing unnecessary medical interventions. Both sensitivity and specificity were calculated along with their 95% confidence intervals.
Calibration Curve: The calibration curve assesses the consistency between predicted probabilities and actual occurrence frequencies. The calibration curve was constructed by dividing samples into bins based on predicted probabilities and comparing the mean predicted probability within each bin to the actual positive rate. A perfect calibration curve would align with the 45° reference line, indicating accurate probability predictions. Deviations above or below this line indicate under-prediction or over-prediction by the model, respectively.

## Results

### Establishment and diagnostic performance evaluation of radiomics diagnostic model

A total of 852 radiomics features were extracted from preoperative contrast-enhanced CT images. These features belong to original and filtered feature classes, including shape features, first-order statistics, and high-order texture features (GLCM, GLRLM, GLSZM, GLDM, NGTDM), applied on original images and wavelet-transformed images. No metabolic parameters were included as only CT data were analyzed.

To prevent overfitting and improve model stability, 10-fold cross-validation was employed. The results of cross-validation, shown in Figure 3(B), indicate that the model stability is highest when Log lambda values range between −4 and −3. At this optimal range, the model achieved the best performance with minimal overfitting. Among the 852 extracted radiomics features, only 13 features were retained after applying the LASSO regression model. The corresponding feature values for these 13 selected features are presented in Figure 3(B), and these features were found to provide the most informative contribution to the model's predictive performance. The specific radiomics features used in model construction are listed in Table 2.

**Figure 3:**
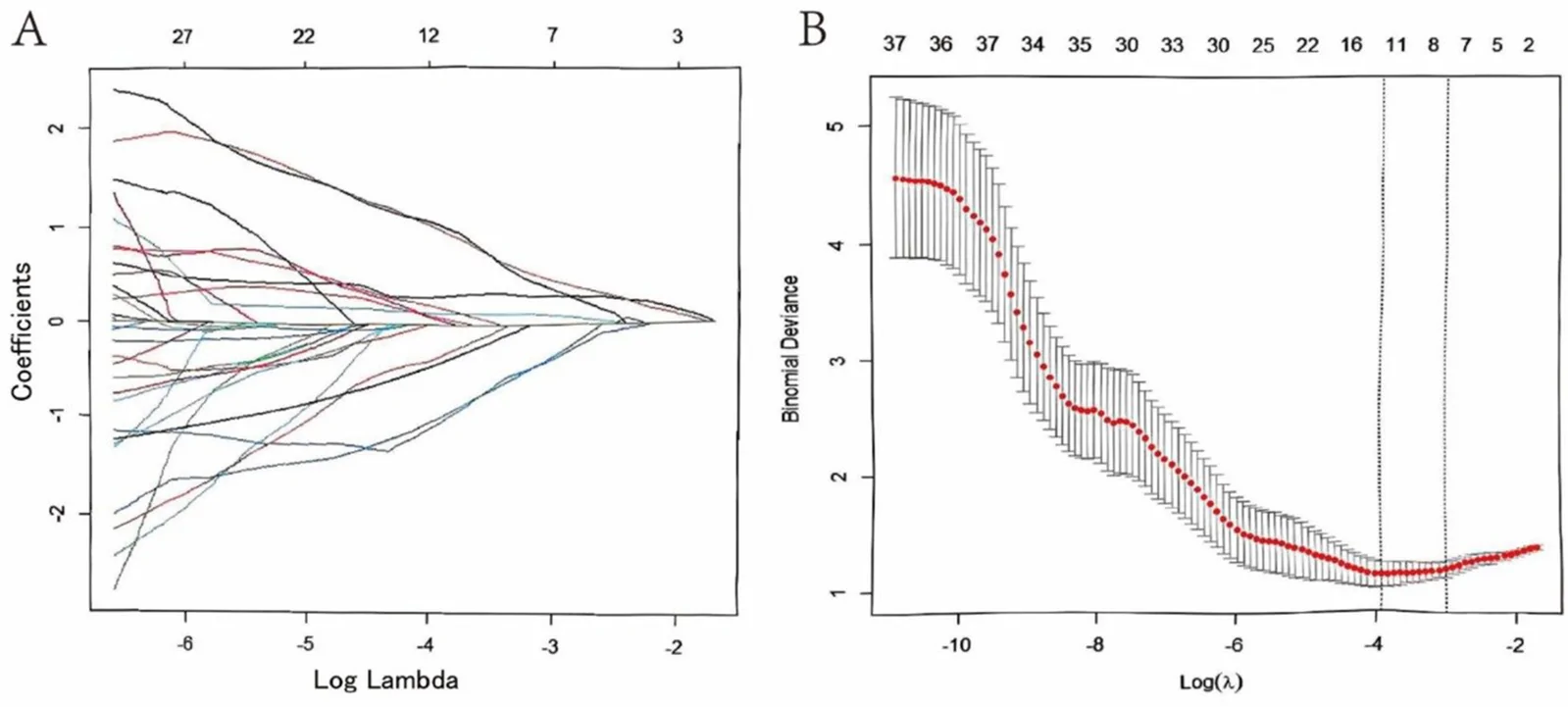
**(A)** LASSO coefficient profiles of radiomics features. **(B)** Cross-validation for optimal feature selection in LASSO regression.

**Table 2:** Radiomics features used in model construction.

Variable	Coefficient
(Intercept)	0.4454545
Original_Shape_VoxelVolume	0.03326619
Original_firstorder_Energy	0.1175763
Original_firstorder_TotalEnergy	2.08 × 10^‒9^
wavelet-LLL_glcm_Imc1	−0.2141904
Original_firstorder_Median	0.04388256
wavelet-LLL_firstorder_Minimum	0.1751349
wavelet-LLH_firstorder_Median	0.02722881
wavelet-HHH_glcm_Imc1	−0.1780711
wavelet-LHH_firstorder_Minimum	−0.01796191
wavelet-LLH_firstorder_Minimum	−0.06671579
wavelet-LLL_glcm_ClusterShade	-0.05711
wavelet-LLH_glcm_Imc1	0.02625127

#### Clinical baseline data

A comparison of baseline characteristics between the high-risk and low-risk groups within both the training and validation cohorts is presented in Table 3. No statistically significant differences were observed in age, sex distribution, smoking history, or BMI between high-risk and low-risk patients in either cohort (all *P* > 0.05), as determined by *t*-tests for continuous variables and chi-square tests for categorical variables.

**Table 3:** Baseline clinical data and tumor marker information of enrolled patients.

Variable	Experimental group (*n* = 110)			Validation group (*n* = 47)		
	Low-risk group (*n* = 61)	High-risk group (*n* = 49)	*P*	Low-risk group (*n* = 26)	High-risk group (*n* = 21)	*P*
Clinical characteristics						
Age (years)	55.82 ± 11.13	55.71 ± 12.09	0.962	55.96 ± 12.09	56.14 ± 14.22	0.963
Gender, *n* (%)			0.207			0.181
Male	25 (41.00%)	26 (46.90%)		15 (57.7%)	8 (38.10%)	
Female	36 (59.00%)	23 (53.10%)		11 (42.3%)	13 (61.90%)	
Smoking history, *n* (%)			0.256			0.070
No	47 (77.00%)	33 (67.30%)		23 (88.50%)	14 (66.77%)	
Persistent or had	14 (23.00%)	16 (32.70%)		3 (11.50%)	7 (33.33%)	
BMI (kg/m²)	24.66 ± 3.25	24.59 ± 3.22	0.909	24.09 ± 2.74	25.24 ± 3.71	0.242
Maximum tumor diameter (cm)	4.00 (2.70,5.50)	5.00 (3.05,7.25)	0.020	4.00 (3.00,5.43)	5.70 (4.3,7.00)	0.031
Tumor markers						
CA125	10.26 (7.21,12.03)	11.2 (9.24,13.01)	0.060	11.20 (10.19,11.20)	11.20 (11.20,11.20)	0.221
CA19-9	9.11 (6.28,12.04)	11.3 (6.44,11.30)	0.042	11.30 (6.47,11.30)	11.30 (10.78,11.30)	0.312
CYFRA21-1	2.10 (1.78,2.10)	2.10 (2.10,2.10)	0.039	2.05 (1.56,2.12)	2.30 (2.06,2.42)	0.007
NSE	13.56 (11.44,15.00)	14.30 (12.58,14.30)	0.131	14.55 (13.53,16.12)	14.30 (12.54,15.10)	0.469
CEA	1.29 (0.85,2.04)	1.76 (1.13,1.93)	0.277	1.77 (0.80,2.32)	1.37 (0.79,2.15)	0.724
AFP	2.64 (2.07,4.96)	3.12 (2.05,6.87)	0.469	2.48 (1.86,5.67)	2.80 (2.28,3.58)	0.781
SCC-Ag	0.90 (0.67,1.05)	1.05 (0.97,1.05)	<0.001	1.05 (0.83,1.22)	1.05 (0.86,1.10)	0.869

Baseline clinical data and tumor marker information of enrolled patients, showing the comparison of clinical characteristics and tumor marker levels between high-risk and low-risk groups. Data are presented as mean ± SD, median (IQR), or *n* (%), as appropriate. *P* values compare low-risk and high-risk groups within each cohort.

However, the maximum tumor diameter was significantly larger in the high-risk group compared to the low-risk group in both the training cohort (median: 5.00 vs. 4.00 cm, *P* = 0.020) and the validation cohort (median: 5.70 vs. 4.00 cm, *P* = 0.031), as assessed by the Mann–Whitney U test. Additionally, in the training cohort, the high-risk group showed significantly higher levels of CA19-9 (*P* = 0.042), CYFRA21-1 (*P* = 0.039), and SCC-Ag (*P* < 0.001) compared to the low-risk group. In the validation cohort, only CYFRA21-1 levels were significantly elevated in the high-risk group (*P* = 0.007).

There were no statistically significant differences in the levels of CA125, NSE, CEA, or AFP between high- and low-risk groups in either cohort (*P* > 0.05).

### Pathological composition of the study cohort

The study cohort encompassed a broad spectrum of mediastinal pathological entities. Among the 157 patients, 70 (44.6%) had thymomas, 30 (19.1%) had thymic carcinoma or other epithelial malignancies, 29 (18.5%) had cystic or non-neoplastic lesions, 15 (9.6%) had neurogenic tumors, 5 (3.2%) had germ-cell tumors, 4 (2.5%) had other mesenchymal tumors, and 4 (2.5%) had other or pathologically indeterminate lesions. Among thymomas, type AB was the most frequent subtype (*n* = 19), followed by B1 (*n* = 9), B2 (*n* = 9), and B3-predominant thymomas (*n* = 8). The B3 category included six pure type B3 thymomas and two mixed thymomas in which the B3 component was predominant. Mixed thymomas were otherwise reported separately in Supplementary Table S1 according to their histological components.

### Establishment of the comprehensive diagnostic model and evaluation of its diagnostic performance

A comprehensive diagnostic model was developed using binary logistic regression based on tumor size, CYFRA21-1, SCC-Ag, CA19-9, and Radscore. To ensure model stability and reduce overfitting, 10-fold cross-validation was applied. The model score was calculated as a linear combination of the regression coefficients and corresponding variables:

Comscore=−7.690+0.116×Size(cm)+0.612×CYFRA21-1+10.086×Radscore+0.847×SCC−Ag+0.027×CA19-9

Each variable in the model corresponds to the regression coefficient, which was derived based on its contribution to the prediction of tumor malignancy. The variables were selected based on statistical significance (*P* < 0.05) from univariate analysis (shown in Table 3) and their clinical relevance, including the reported association of CYFRA21-1 with several epithelial malignancies.

To assess model performance, 10-fold cross-validation was conducted by dividing the data into 10 subsets. Nine subsets were used for training, and the remaining one for validation. This process was repeated 10 times, and the average performance metrics were calculated to evaluate the model's robustness and generalizability.

Next, nomogram were generated based on the integrated model to visually illustrate the relationship between the selected variables and the predicted high tumor risk (aggressiveness/malignant potential) (Figure 4).

**Figure 4:**
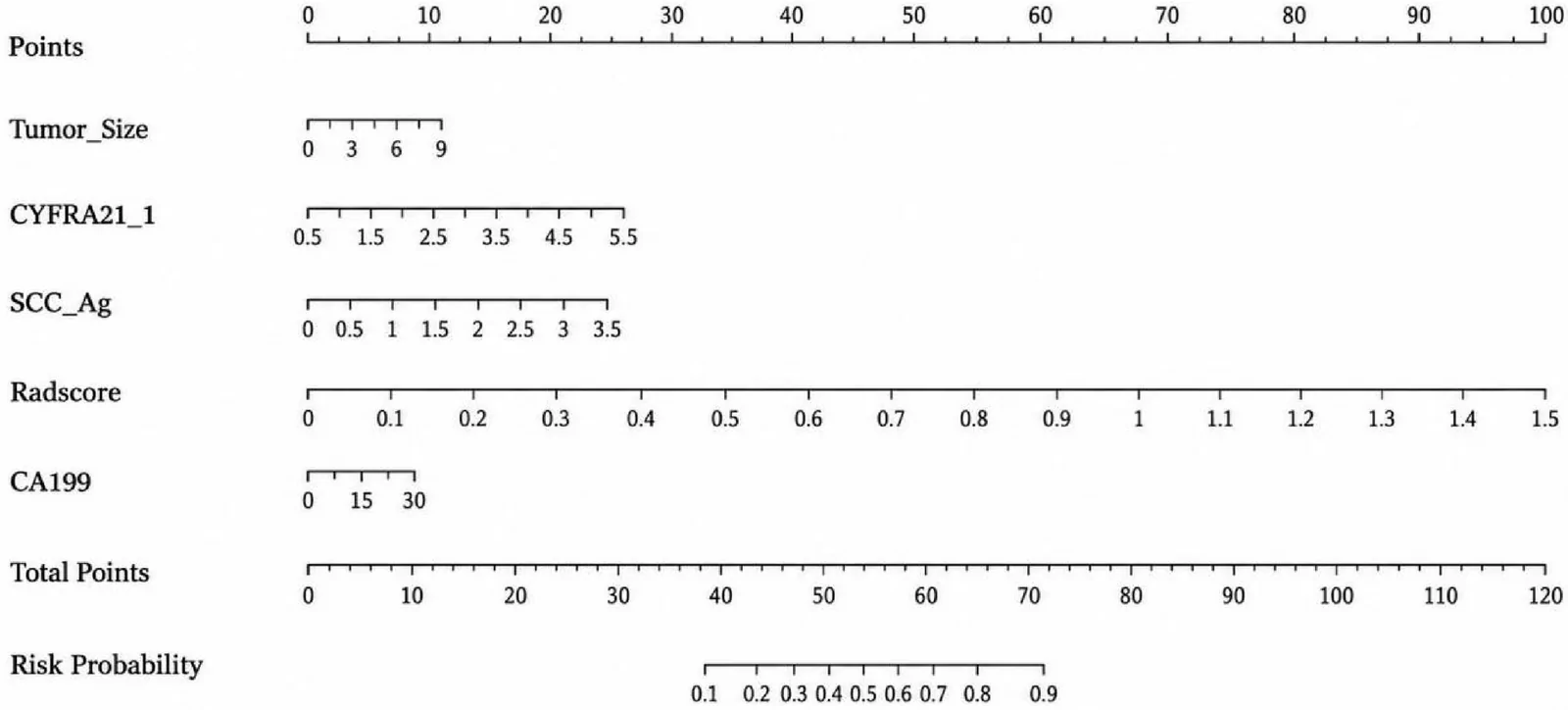
Nomogram showing the use of the integrated model to predict high-risk lesions.

#### Comparison of the two models

The diagnostic performance of the radiomics and integrated diagnostic models is summarized in Table 4, and the corresponding ROC curves are shown in Figures 5(A,B).

**Table 4:** Diagnostic performance of each diagnostic model in the experimental group and the validation group.

Model	AUC (95%CI)	SEN	SPEC
Experimental group			
Radiomics diagnostic model	0.8985 (0.8407–0.9562)	0.8197	0.8571
Comprehensive diagnostic model	0.9117 (0.8586–0.9648)	0.8525	0.8163
Validation group			
Radiomics diagnostic model	0.8599 (0.7488–0.9709)	0.8077	0.7619
Comprehensive diagnostic model	0.8718 (0.7739–0.9697)	0.8462	0.7143

**Figure 5:**
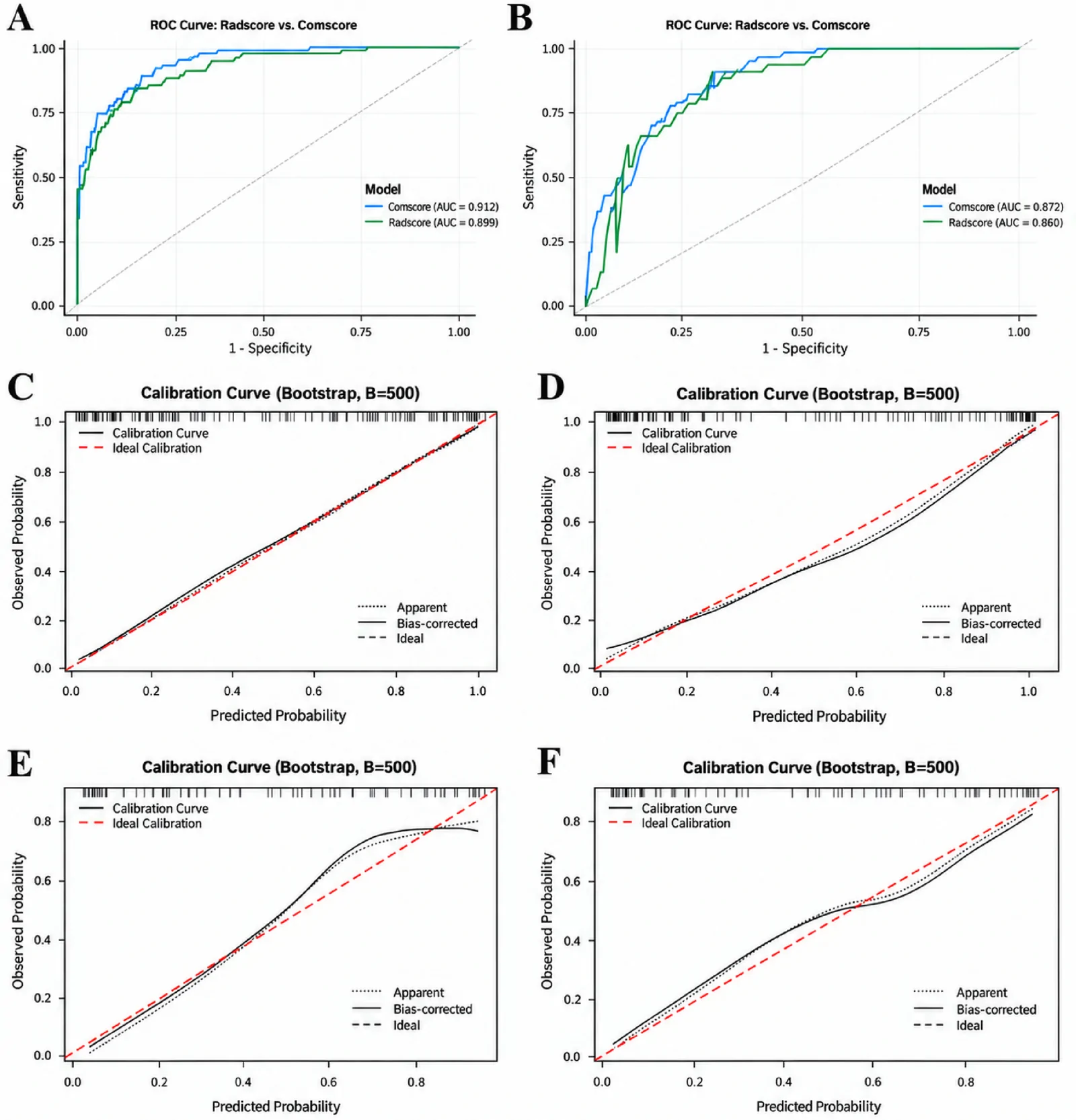
Diagnostic performance and calibration curves of the radiomics and comprehensive models. **(A,B)** ROC curves of the radiomics and comprehensive models in the experimental and validation cohorts, respectively. **(C)** Calibration curve of the radiomics model in the training cohort. **(D)** Calibration curve of the comprehensive model in the training cohort. **(E)** Calibration curve of the radiomics model in the validation cohort. **(F)** Calibration curve of the comprehensive model in the validation cohort.

In the training cohort, the radiomics model had an AUC of 0.8985 (95% CI: 0.8407–0.9562), with a sensitivity of 81.97% and a specificity of 85.71%; while the AUC value for the integrated model was 0.9117 (95% CI: 0.8586–0.9648), with a sensitivity of 85.25% and a specificity of 81.63%.

In the validation cohort (*n* = 47), the radiomics model had an AUC of 0.8599 (95% CI: 0.7488–0.9709), with a sensitivity of 80.77% and a specificity of 76.19%; while the AUC value for the integrated model was 0.8718 (95% CI: 0.7739–0.9697), with a sensitivity of 84.62% and a specificity of 71.43%.

To statistically compare the performance of the two models, a DeLong test was conducted on the validation cohort. The results showed no significant difference between the AUC values of the integrated model and the radiomics model (*Z* = −1.1643, *P* = 0.2443), suggesting that the improvement in the AUC value of the integrated model is not yet statistically significant. Therefore, the relative advantage of the integrated model should be interpreted with caution—although it demonstrated numerically higher AUC values in both the training and validation cohorts, the current evidence does not support the conclusion that the integrated model is statistically significantly superior to the radiomics model. This result may be related to the limited sample size of this study; future research in larger cohorts is needed to further validate whether multimodal integration can indeed provide incremental diagnostic value beyond that of single-modality radiomics.

Although no statistically significant difference was observed, the integrated model may still offer practical advantages—as it simultaneously integrates radiomics features and clinical variables, thereby enhancing clinical interpretability and real-world applicability. The model demonstrated balanced diagnostic performance (with a sensitivity of 84.62% and a specificity of 71.43% in the validation cohort), indicating that it can serve as a reliable clinical decision-support tool for evaluating mediastinal lesions. In preoperative risk screening, improving sensitivity for high-risk lesions is typically considered a primary goal; by incorporating serum biomarkers, this integrated model achieves this objective, fully demonstrating the clinical value of multimodal data fusion.

However, the model's specificity is relatively moderate, suggesting that caution should be exercised when applying it to low-risk populations. For patients classified as high-risk by this model (including those with malignant and invasive thymomas), a multidisciplinary consultation is recommended to confirm the diagnosis and guide clinical management; conversely, patients classified as low-risk may benefit from short-term imaging follow-up to minimize the risk of missed diagnosis.

Furthermore, Figures 5(C–F) displays the calibration curves for both models, indicating that both models demonstrate good consistency.

### Decision curve analysis

Decision curve analysis was further performed to assess the potential clinical utility of the prediction models (Figure 6). In both the training and internal validation cohorts, the integrated model generally provided a positive net benefit across a broad range of threshold probabilities and showed greater clinical net benefit than the default treat-all and treat-none strategies over most clinically relevant thresholds. The radiomics model also demonstrated a favorable net benefit across a substantial range of threshold probabilities, whereas the clinical model showed comparatively less stable net benefit, particularly at intermediate threshold probabilities. Overall, these findings suggest that the integrated model may provide clinically useful decision support for preoperative risk stratification, although its incremental clinical benefit over the radiomics-only model was not uniform across all threshold probabilities.

**Figure 6:**
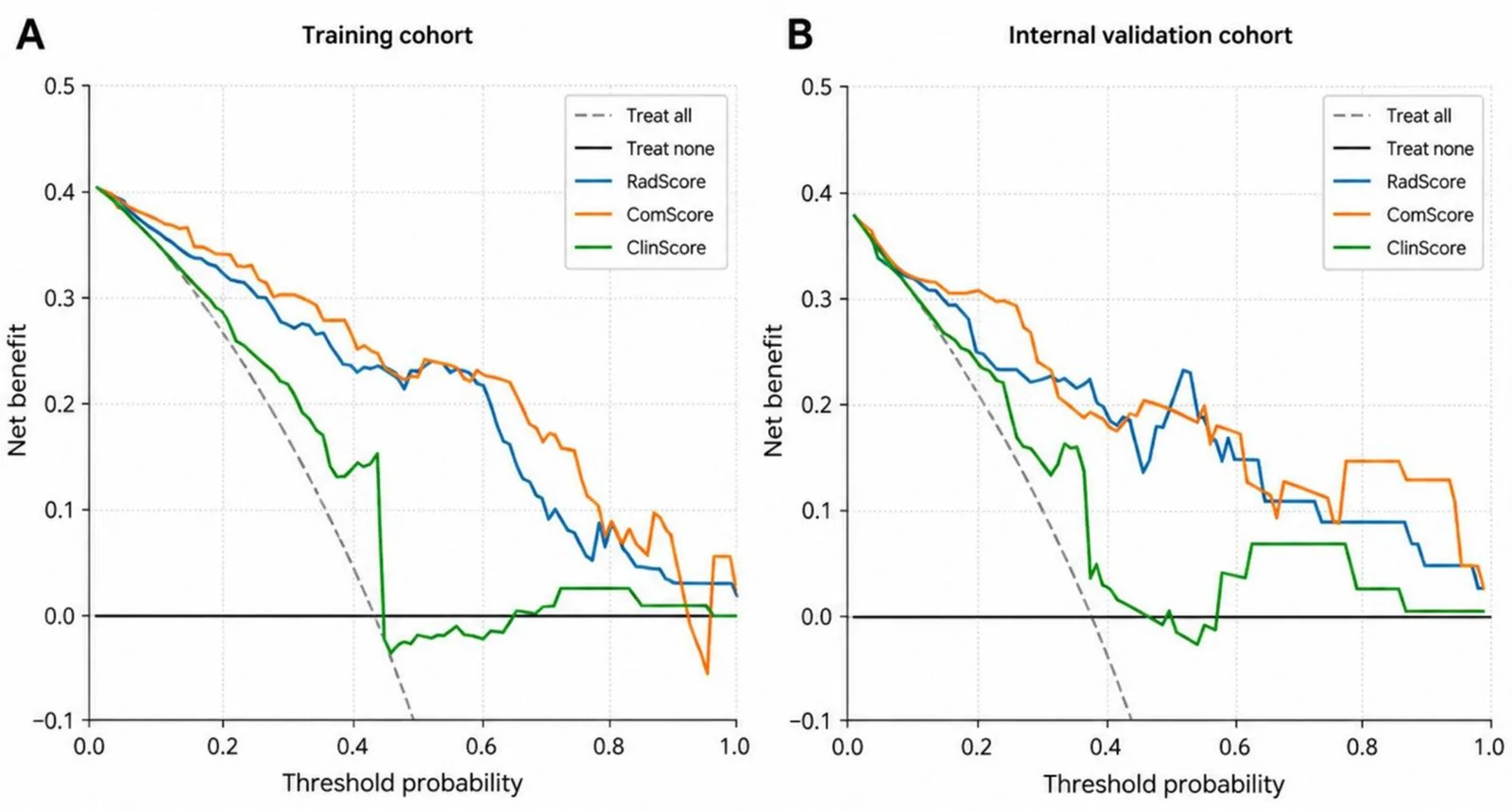
Decision curve analysis of the prediction models: **(A)** decision curve analysis in the training cohort. **(B)** Decision curve analysis in the internal validation cohort. The *x*-axis represents the threshold probability, and the *y*-axis represents the net benefit. The black horizontal line indicates the “treat none” strategy, whereas the gray dashed line represents the “treat all” strategy. Radscore represents the radiomics model, ClinScore represents the clinical model, and Comscore represents the integrated radiomics-clinical model.

To evaluate the performance advantages of the comprehensive model relative to existing methods, this study conducted an indirect comparison with the following two simplified reference models: (1) a logistic regression model based on clinical variables (tumor size + serum tumor markers); (2) a clinical interpretation model based on CT morphological features (border clarity, enhancement degree, presence or absence of cystic necrosis, etc.) (independently assessed by two radiologists). In the validation cohort, the AUC of the clinical variable model was 0.8234 (95% CI: 0.7123–0.9345), the mean AUC of the CT morphological model was 0.8017 (95% CI: 0.6935–0.9099), while the AUC of the integrated model proposed in this study was 0.8718, which was numerically superior to the two reference models mentioned above.

## Discussion

### Research significance and main findings

Mediastinal tumors pose significant diagnostic challenges due to their complex pathological characteristics and nonspecific clinical manifestations (18, 19). Conventional CT imaging often fails to reliably distinguish between benign and malignant lesions, particularly when lesion characteristics overlap (8, 20). In this study, we developed a predictive model that combines CT radiomics features with clinical factors—the tumor markers CYFRA21-1, CA19-9, and SCC-Ag, as well as tumor size—to improve the accuracy of stratifying high-risk and low-risk lesions.

CYFRA21-1 is a soluble fragment of cytokeratin 19. In epithelial-origin malignancies such as lung cancer (particularly non-small cell lung cancer), there is substantial research evidence supporting its association with tumor burden and prognosis (21, 22). In the field of mediastinal tumors, evidence for the clinical application of CYFRA21-1 is still accumulating. This study found that CYFRA21-1 was significantly associated with the high-risk group in both the training and validation cohorts, suggesting that it may serve as an auxiliary reference indicator for high-risk mediastinal lesions. However, its independent diagnostic value in mediastinal tumors still requires validation through studies with larger samples and cohorts; at this stage, it should not be characterized as a definitive diagnostic biomarker for mediastinal tumors.

SCC-Ag has been investigated as a serum marker in thymic carcinoma. In the NEJ023 cohort, its prognostic association was observed in selected histological analyses, although its diagnostic utility for mediastinal tumors remains uncertain (23). Previous studies have demonstrated its usefulness in identifying mediastinal squamous carcinomas. In our study, SCC-Ag demonstrated strong discriminatory power in the experimental cohort, but its performance in the validation cohort was less consistent. This discrepancy may be due to the smaller proportion of squamous tumors in the validation cohort, underscoring the importance of tumor subtype in biomarker selection.

CA19-9, primarily elevated in gastrointestinal malignancies, also serves as a tumor marker in mediastinal lesions (24). While its role in preoperative diagnosis of mediastinal tumors appears to be less consistent than CYFRA21-1 and SCC-Ag, our results show that combining CA19-9 with other clinical and radiomics features improves the overall model performance, reflecting tumor aggressiveness and biological behavior.

The combined model outperformed clinical or radiomics models alone, with higher AUCs in both cohorts, consistent with previous studies emphasizing multi-source data fusion for mediastinal tumor diagnosis.

From a clinical perspective, the proposed model is intended to provide adjunctive quantitative information during the preoperative assessment of mediastinal lesions rather than to independently determine management decisions. Its potential application may be considered in several specific clinical scenarios. First, for patients assigned a high predicted risk, particularly when conventional CT findings are suspicious or the extent of invasion is uncertain, the model output may prompt more comprehensive preoperative evaluation, including additional imaging such as contrast-enhanced MRI or PET/CT when clinically indicated, assessment of resectability, and multidisciplinary discussion. Histological confirmation by image-guided biopsy may be considered when tissue diagnosis is required to guide treatment, particularly in diagnostically uncertain or potentially unresectable lesions; however, biopsy should not be performed solely on the basis of a high-risk model prediction.

Second, for patients assigned a low predicted risk whose conventional imaging findings are also strongly suggestive of a benign or indolent lesion, such as a well-defined homogeneous cystic lesion without enhancement, the model result may provide additional supportive information when clinicians are considering imaging surveillance rather than immediate surgical intervention. Nevertheless, because the present study included only surgically treated patients, the model has not yet been validated as a tool for safely deferring surgery in conservatively managed populations.

Third, the model may be particularly useful in clinically indeterminate or discordant cases in which conventional imaging findings and clinical assessment do not provide a consistent risk estimate. In such situations, the predicted probability may serve as an additional quantitative reference during multidisciplinary and shared decision-making, helping clinicians and patients weigh the potential benefits of further diagnostic evaluation or surgical intervention against the risks of conservative management. Importantly, the model should be regarded as a decision-support tool that complements, rather than replaces, conventional imaging assessment, pathological evaluation, and multidisciplinary clinical judgment. Prospective external validation is required before the model can be incorporated into routine clinical decision pathways.

The methodological contribution of this study should be distinguished from the use of radiomics itself, which has already been extensively investigated in oncologic imaging. The distinctive feature of our approach is the integration of CT-derived radiomics features with serum tumor biomarkers within a single multimodal prediction model. Radiomics features quantitatively characterize tumor morphology, intensity distribution, and intratumoral heterogeneity, whereas serum biomarkers such as CYFRA21-1, SCC-Ag, and CA19-9 provide complementary information related to tumor-associated biological activity. Integrating these heterogeneous sources of information may therefore provide a more comprehensive representation of lesion characteristics than either imaging or serum biomarkers alone. In this context, the present study provides preliminary evidence supporting the feasibility of combining imaging phenotypes and circulating biomarkers for preoperative risk stratification of mediastinal lesions. Nevertheless, because the improvement in AUC over the radiomics-only model was numerical rather than statistically significant in the internal validation cohort, the incremental predictive value of this multimodal strategy should be confirmed in larger, independent, multicenter cohorts.

### Consistent risk factors across cohorts

#### Maximum tumor diameter

This parameter was consistently a statistically significant differentiator between low-risk and high-risk groups in both the experimental (*P* = 0.020) and validation (*P* = 0.031) cohorts. Larger tumors in high-risk patients are consistent with well-established oncological principles that link tumor size with advanced disease, higher pathological stages, increased metastatic potential, and poorer prognosis.

#### CYFRA21-1

CYFRA21-1 is a soluble fragment of cytokeratin 19. In epithelial malignancies, particularly non-small cell lung cancer, substantial evidence supports its association with tumor burden and prognosis. However, evidence supporting its diagnostic application in mediastinal tumors remains limited. In the present study, higher CYFRA21-1 levels were associated with high-risk lesions in both the training and internal validation cohorts, suggesting that CYFRA21-1 may serve as an adjunctive indicator within a multimodal risk-stratification framework. Nevertheless, these findings do not establish its independent diagnostic utility, and CYFRA21-1 should not be regarded as a definitive standalone diagnostic biomarker for mediastinal tumors. Further validation in larger, independent, multicenter cohorts is warranted.

#### SCC-Ag

SCC-Ag levels were significantly elevated in the high-risk group in the experimental cohort (*P* < 0.001), indicating its potential use in risk stratification for squamous cell carcinoma. However, this significant difference was not observed in the validation cohort (*P* = 0.869). This discrepancy may be due to the smaller size of the validation cohort, different proportions of squamous vs. non-squamous cancers, or inherent variability in SCC-Ag levels. While SCC-Ag demonstrated strong discriminatory power in the derivation cohort, its generalizability requires further validation.

#### CA19-9

A significant difference was observed in the experimental cohort, where CA19-9 levels were higher in the high-risk group (*P* = 0.042). However, this difference was not statistically significant in the validation cohort (*P* = 0.312), suggesting that CA19-9 may not be as reliable across different cohorts compared to other biomarkers like CYFRA21-1 and tumor size.

#### Radscore

This line graph identifies Radscore as a key predictive factor (coefficient: 10.086), highlighting its significant contribution to the Comscore. By quantifying textural features, morphological parameters, and heterogeneity indices in CT images, Radscore may indirectly reflect the structural complexity of the tumor. Although previous studies have reported associations between radiomics features and selected histopathological characteristics, their correspondence with specific biological or molecular mechanisms remains insufficiently established and requires further validation through matched imaging–pathology and molecular studies. Therefore, at this stage, Radscore should be regarded as a quantitative radiological indicator reflecting tumor imaging heterogeneity rather than a direct biological surrogate marker.

### Innovations: synergistic value of integrating serum biomarkers with radiomics

The main innovation of this study lies in the integration of serum tumor markers and CT radiomics features, which can produce complementary and synergistic effects in distinguishing high-risk from low-risk mediastinal lesions. Compared with previous studies that were largely limited to imaging features or single clinical parameters, this model systematically combines the ability of radiomics to quantify macroscopic heterogeneity with the microscopic molecular information provided by serum biomarkers, thereby establishing a multimodal, multiscale integrated predictive strategy.

#### Multimodal feature fusion

Radiomics enables the quantification of tumor microheterogeneity and has shown significant promise in oncologic diagnosis (25). However, its standalone diagnostic power remains limited, particularly for complex mediastinal lesions. Radiomics primarily captures the macroscopic morphological heterogeneity of tumors, including features such as texture and shape. In contrast, serum biomarkers such as CYFRA21-1 and SCC-Ag provide insight into microscopic molecular activities, including cellular proliferation and apoptosis in epithelial-derived cells. By integrating these two sources of information-macroscopic features from radiomics and microscopic molecular data from biomarkers-the proposed model constructs a more comprehensive biological profile of tumors.

As demonstrated by the results, this fusion consistently improves diagnostic performance. Notably, CYFRA21-1 emerged as a significant predictor across both cohorts, providing robust incremental value to the radiomics-based model. This integration enhances the model's ability to differentiate between benign and malignant lesions, further underscoring the importance of combining imaging data with molecular markers for more accurate tumor assessment.

#### Clinical realism of study population

Unlike studies focusing on specific lesions, such as thymomas or cysts, this research included a broader range of mediastinal masses—both benign and malignant, solid and cystic—more accurately reflecting the complexity of real-world clinical scenarios. This approach enhances the model's generalizability and applicability to a wide variety of clinical situations (26, 27).

#### Integration of statistical evidence and clinical relevance

Variable selection for the integrated model was guided by both statistical evidence from the training cohort and clinical relevance. Tumor size, CYFRA21-1, SCC-Ag, and CA19-9 showed statistically significant differences between the high- and low-risk groups in the training cohort and were therefore considered candidate clinical predictors. These variables were subsequently integrated with the radiomics score to construct the final prediction model. Although the associations of individual serum biomarkers were not uniformly reproduced in the internal validation cohort, their combined contribution within the integrated model may reflect complementary biological information beyond imaging-derived features alone. Nevertheless, the predictive contribution of these biomarkers, particularly those showing inconsistent associations across cohorts, should be further evaluated in larger independent datasets (28, 29).

#### Clear technical positioning

Radiomics was employed as the primary tool for feature extraction, with logistic regression applied to construct the model. This traditional machine learning approach strikes a balance between diagnostic accuracy and interpretability, which is critical for clinical translation. The integration of multiple data types into a robust and transparent framework allows the model to address the diagnostic challenges presented by the high heterogeneity of mediastinal tumors (29).

### Limitations and future directions

#### Subjectivity in ROI segmentation and limited feature interpretability

This study relied on manual delineation of regions of interest (ROIs), which, although performed by experienced radiologists, may introduce variability and impact the reproducibility of feature extraction. Furthermore, while the nomogram provides an intuitive visualization, the biological significance of radiomics features may not be immediately apparent to clinicians. Future studies should integrate deep learning-assisted segmentation techniques and focus on enhancing model interpretability to foster greater clinical confidence and facilitate broader clinical adoption (29).

#### Limited sample size and lack of external validation

Limited sample size, lack of external validation, and selection bias in the surgical population. This study was a single-center retrospective study with a relatively small sample size (*n* = 157); although internal validation was performed using Lasso regression and 10-fold cross-validation, there remains a risk of overfitting. Furthermore, this study included only patients who underwent surgical resection and had a definitive pathological diagnosis; it does not represent the population of patients with mediastinal masses who underwent conservative follow-up or only needle biopsy. This selection bias may limit the model's generalizability to non-surgical populations. Finally, this study did not conduct external validation, and the model's generalizability remains to be confirmed by an independent external dataset. Increasing the sample size and conducting multicenter external validation are crucial for enhancing the model's robustness and generalizability (30, 31).

#### Potential bias in variable selection and preoperative interventions

Although LASSO regression was employed for feature selection, some important clinical variables may have been excluded. Additionally, preoperative image-guided biopsies performed in certain cases may have altered tumor characteristics, potentially introducing selection bias. Future studies should account for such confounding factors to improve the accuracy and reliability of the model (30, 32).

#### Limited heterogeneity in pathological subtypes

Although the present cohort included a broad spectrum of mediastinal pathological entities, their distribution was imbalanced. Thymomas and epithelial malignancies accounted for the majority of cases, whereas several uncommon pathological subtypes were represented by only a small number of patients. Such spectrum imbalance may influence model performance and limit the reliability of subtype-specific predictions. Given the limited number of cases within individual pathological subgroups, subtype-specific diagnostic performance was not evaluated in the present study. Future multicenter studies with larger and more balanced pathological cohorts are warranted to assess model performance across individual histological subtypes and to explore subtype-specific predictive models (15, 19).

#### Future research and clinical translation strategies

Prospective Multicenter Validation: Rigorous external validation across multiple centers with predefined protocols is essential for regulatory approval and clinical adoption.

Technical Optimization: The integration of automated segmentation techniques and standardization of features across various imaging platforms can improve the robustness of the model and reduce dependency on operator skill.

Multimodal and Subtype-Specific Modeling: Incorporating genomics, proteomics, and other omics data will enable more precise molecular-level predictions. Additionally, subtype-specific models can improve performance across heterogeneous tumor types, offering more tailored diagnostic insights.

Development of Clinical Decision-Support Tools: User-friendly platforms embedded within Picture Archiving and Communication Systems (PACS) or Electronic Medical Record (EMR) systems could facilitate the real-world implementation of the model.

Implementation Science and Cost-Effectiveness Analysis: Future studies should assess the real-world feasibility, clinician acceptance, clinical impact, and economic value of model deployment, ensuring that the model's adoption is both clinically and economically viable (33).

#### Selection bias in the surgical population

This study included only patients with mediastinal tumors who underwent surgical resection and received a definitive postoperative pathological diagnosis; therefore, the study cohort does not represent the full spectrum of mediastinal masses—particularly small lesions or benign lesions with typical imaging features that are typically managed with conservative follow-up rather than surgical intervention. This inclusion strategy may limit the model's applicability to non-surgical populations (such as patients with incidentally detected small lesions identified during outpatient visits, elderly patients, or those with poor surgical tolerance). Future studies should include patients with mediastinal masses who undergo needle biopsy or regular imaging follow-up to assess the model's generalizability in broader clinical settings.

## Conclusion

We developed a multimodal model that integrates CT radiomics with clinical indicators, including serum tumor markers, for the preoperative stratification of high-risk and low-risk mediastinal lesions. The integrated model demonstrated numerically higher AUC values than the radiomics-only model in both the training and internal validation cohorts. However, the difference in AUC between the two models did not reach statistical significance in the internal validation cohort (DeLong test, *P* = 0.2443). Therefore, the observed improvement in model performance should be interpreted as numerical rather than statistically confirmed. Given the single-center retrospective design and the lack of external validation, the potential incremental value and generalizability of the integrated model require further validation in larger, independent, multicenter cohorts.

## Data Availability

The original contributions presented in the study are included in the article/Supplementary Material, further inquiries can be directed to the corresponding author.
