# Supplementary material for: Preoperative risk stratification of mediastinal masses using a predictive model based on CT radiomics and clinical data
Source: Front Surg. 2026 Sep 17;13:1937652. doi: 10.3389/fsurg.2026.1937652 (PMC13627096; doi:10.3389/fsurg.2026.1937652)
Supplement: Supplementary file 1 [file Table1.docx]

**Supplementary Table S1. Distribution of pathological diagnoses in the training, validation, and overall cohorts**

| **Pathological category / histopathological subtype** | **Training cohort (n = 110), n (%)** | **Validation cohort (n = 47), n (%)** | **Overall cohort (n = 157), n (%)** |
| --- | --- | --- | --- |
| **Thymoma, subtotal** | **44 (40.0)** | **26 (55.3)** | **70 (44.6)** |
| Type A | 2 (1.8) | 1 (2.1) | 3 (1.9) |
| Type AB | 16 (14.5) | 3 (6.4) | 19 (12.1) |
| Type B1 | 6 (5.5) | 3 (6.4) | 9 (5.7) |
| Type B2 | 3 (2.7) | 6 (12.8) | 9 (5.7) |
| Type B3 | 5 (4.5) | 1 (2.1) | 6 (3.8) |
| Mixed B1/B2 | 1 (0.9) | 3 (6.4) | 4 (2.5) |
| Mixed B1/B3 | 1 (0.9) | 0 (0.0) | 1 (0.6) |
| Mixed B2/B3 | 2 (1.8) | 3 (6.4) | 5 (3.2) |
| Mixed AB/B2 | 1 (0.9) | 0 (0.0) | 1 (0.6) |
| Micronodular thymoma with lymphoid stroma | 1 (0.9) | 2 (4.3) | 3 (1.9) |
| Thymoma | 6 (5.5) | 4 (8.5) | 10 (6.4) |
| **Carcinoma/malignant epithelial tumor, subtotal** | **19 (17.3)** | **5 (10.6)** | **24 (15.3)** |
| Squamous cell carcinoma | 15 (13.6) | 5 (10.6) | 20 (12.7) |
| Thymic carcinoma | 1 (0.9) | 0 (0.0) | 1 (0.6) |
| Micronodular thymic carcinoma | 1 (0.9) | 0 (0.0) | 1 (0.6) |
| Papillary carcinoma | 1 (0.9) | 0 (0.0) | 1 (0.6) |
| Poorly differentiated carcinoma | 1 (0.9) | 0 (0.0) | 1 (0.6) |
| **Neuroendocrine tumor, subtotal** | **3 (2.7)** | **2 (4.3)** | **5 (3.2)** |
| Atypical carcinoid / neuroendocrine tumor G2 | 2 (1.8) | 2 (4.3) | 4 (2.5) |
| Neuroendocrine carcinoma | 1 (0.9) | 0 (0.0) | 1 (0.6) |
| **Cystic/non-neoplastic lesion, subtotal** | **23 (20.9)** | **6 (12.8)** | **29 (18.5)** |
| Thymic cyst | 11 (10.0) | 0 (0.0) | 11 (7.0) |
| Bronchogenic cyst | 8 (7.3) | 2 (4.3) | 10 (6.4) |
| Mesothelial cyst | 0 (0.0) | 2 (4.3) | 2 (1.3) |
| Pericardial cyst | 0 (0.0) | 1 (2.1) | 1 (0.6) |
| Parathyroid cyst | 1 (0.9) | 0 (0.0) | 1 (0.6) |
| Thymic lymphoid hyperplasia | 1 (0.9) | 0 (0.0) | 1 (0.6) |
| Thymic lymphoid follicular hyperplasia with cyst formation | 1 (0.9) | 0 (0.0) | 1 (0.6) |
| Thymic hyperplasia with cystic change | 0 (0.0) | 1 (2.1) | 1 (0.6) |
| Well-differentiated glands and cyst wall | 1 (0.9) | 0 (0.0) | 1 (0.6) |
| **Neurogenic tumor, subtotal** | **9 (8.2)** | **6 (12.8)** | **15 (9.6)** |
| Schwannoma | 6 (5.5) | 5 (10.6) | 11 (7.0) |
| Cellular schwannoma | 1 (0.9) | 0 (0.0) | 1 (0.6) |
| Atypical schwannoma | 0 (0.0) | 1 (2.1) | 1 (0.6) |
| Paraganglioma | 1 (0.9) | 0 (0.0) | 1 (0.6) |
| Neurogenic tumor | 1 (0.9) | 0 (0.0) | 1 (0.6) |
| **Germ-cell tumor, subtotal** | **4 (3.6)** | **1 (2.1)** | **5 (3.2)** |
| Mature teratoma | 3 (2.7) | 1 (2.1) | 4 (2.5) |
| Seminoma | 1 (0.9) | 0 (0.0) | 1 (0.6) |
| **Mesenchymal tumor, subtotal** | **4 (3.6)** | **0 (0.0)** | **4 (2.5)** |
| Cavernous hemangioma | 1 (0.9) | 0 (0.0) | 1 (0.6) |
| Smooth muscle tumor | 1 (0.9) | 0 (0.0) | 1 (0.6) |
| Alveolar soft-part sarcoma | 1 (0.9) | 0 (0.0) | 1 (0.6) |
| Spindle cell tumor | 1 (0.9) | 0 (0.0) | 1 (0.6) |
| **Other/indeterminate lesion, subtotal** | **4 (3.6)** | **1 (2.1)** | **5 (3.2)** |
| Malignant tumor | 1 (0.9) | 1 (2.1) | 2 (1.3) |
| Thymic epithelial tumor | 1 (0.9) | 0 (0.0) | 1 (0.6) |
| Thymic malignant tumor | 1 (0.9) | 0 (0.0) | 1 (0.6) |
| Round-cell malignant tumor | 1 (0.9) | 0 (0.0) | 1 (0.6) |
| **Total** | **110 (100.0)** | **47 (100.0)** | **157 (100.0)** |
